# *UBE3A* Inhibits Trophoblast Cell Migration and Invasion by Promoting *ITGB1* Degradation and Affecting *PI3K/AKT* Signaling

**DOI:** 10.1002/kjm2.70122

**Published:** 2025-10-15

**Authors:** Xiu‐Jun Li, Ning Wang, Fan Jiang, Jie‐Cheng Yang, Yan‐Yun Wang, Kun Wang

**Affiliations:** ^1^ Department of Obstetrics Affiliated Hospital of Hebei Engineering University Handan City China; ^2^ College of the Environment and Health Yanching Institute of Technology Langfang City China; ^3^ Child Care Merice Cody Public School Toronto Canada; ^4^ Department of Obstetrics Handan Central Hospital Handan City China

**Keywords:** ITGB1, PI3K/AKT signaling, preeclampsia, UBE3A

## Abstract

Preeclampsia (PE) is an obstetric disease that is characterized by reduced migration and invasion of placental trophoblast cells. Here, the effects of the E3 ubiquitin ligase UBE3A on the migration and invasion of trophoblast cells were evaluated. RT‐qPCR and Western blotting were used to measure the expression of genes and proteins. Immunohistochemical (IHC) staining was used to determine UBE3A and ITGB1 levels in the placental tissues of PE patients. Cell viability was evaluated with a CCK‐8 assay. Wound healing and Transwell assays were used to evaluate cell migration and invasion, respectively. Cell cycle progression and apoptosis were analyzed by flow cytometry. Co‐immunoprecipitation (Co‐IP) was used to verify molecular interactions. Our results revealed that the mRNA and protein levels of UBE3A were upregulated, whereas ITGB1 expression was downregulated in the placental tissues of PE patients. Depletion of UBE3A promoted the migration and invasion of HTR‐8/SVneo cells while inhibiting apoptosis. The phosphorylation of PI3K and AKT increased after UBE3A was silenced. Mechanistically, UBE3A induced the ubiquitination and degradation of ITGB1. Functionally, UBE3A reduced cell migration and invasion, as well as induced apoptosis by negatively regulating ITGB1 mediating PI3K/AKT signaling. In summary, our results revealed that UBE3A hindered the migration and invasion of trophoblast cells by facilitating ITGB1 degradation and affecting PI3K/AKT signaling, providing a new therapeutic target for PE treatment.

**Abbreviations**
CCK‐8: cell counting kit‐8
CHX: cycloheximide
Co‐IP: co‐immunoprecipitation
CST: cell signaling technology
DAB: diaminobenzidine
FBS: fetal bovine serum
IHC: immunohistochemical
IP: immunoprecipitation
ITGB1: integrin β1
PBS: phosphate‐buffered saline
PE: preeclampsia
PI: propidium iodide
PI3K: phosphatidylinositol 3‐kinase
SD: standard deviation
UBE3A: ubiquitin protein ligase E3A
UPS: ubiquitin‐proteasome system

## 1 Introduction

Preeclampsia (PE) is a form of pregnancy‐induced hypertension that is defined as hypertension that occurs after 20 weeks of pregnancy, accompanied by maternal organ or uterine placental dysfunction or proteinuria [1]. Worldwide, 3%–5% of pregnant women are affected by PE [2]. PE is a primary factor that contributes to mortality among pregnant women and newborns [3]. Trophoblast cells are specialized placental villous cells that invade and reshape the uterine spiral artery during normal pregnancy, facilitating maternal‐placental blood circulation [4]. Most studies suggest that the main cause of PE is the disordered uterine spiral artery remodeling and decreased placental perfusion that are caused by insufficient proliferation, migration, and invasion of trophoblast cells [5]. Therefore, exploring and identifying the mechanisms that affect the function of trophoblast cells may provide promising targets for the treatment of PE.

The ubiquitin‐proteasome system (UPS) participates in modulating the degradation of proteins in organisms, thus maintaining the homeostasis of proteins and cells, and it plays a key role in cell growth and development [6]. Ubiquitination is a prevalent and complex posttranslational protein modification process that is very common in the UPS system. Ubiquitination involves a three‐level enzyme‐linked reaction in which ubiquitin‐activating enzyme E1, ubiquitin‐binding enzyme E2, and ubiquitin ligase E3 participate [7]. Ubiquitination plays a vital role in many physiological processes, such as cell survival and innate immunity [8]. A recent study revealed that ubiquitination is closely related to PE regulation [9]. The ubiquitin protein ligase E3A (UBE3A) protein is an important member of the ubiquitin ligase E3 family, and it is responsible for regulating DNA replication, translation, intracellular transport, and centrosome regulation [10]. A previous study revealed that UBE3A expression is elevated in PE [11]. However, the mechanism by which UBE3A regulates trophoblast cell migration and invasion remains unclear.

Integrin β1 (ITGB1) is a transmembrane glycoprotein receptor that plays a crucial role in various physiological and pathological processes [12]. The expression of ITGB1 is downregulated in the placental tissues of PE patients, suggesting the potential effect of ITGB1 on PE [13]. Overexpressed ITGB1 can increase the proliferation, migration, and invasion of HTR‐8/SVneo cells, reduce apoptosis, and increase the phosphorylation of phosphatidylinositol 3‐kinase (PI3K)/AKT [14]. Through bioinformatics prediction, it was found that UBE3A may be important for trophoblast cell migration and invasion by mediating the ubiquitination of ITGB1. Nevertheless, the specific regulatory role remains unclear.

In summary, we propose that UBE3A inhibits the activation of PI3K/AKT signaling by promoting ITGB1 degradation, thereby inhibiting trophoblast cell migration and invasion and promoting PE. Thus, UBE3A may be an inducible protein in PE progression.

## 2 Materials and Methods

### 2.1 Specimen Collection and Processing

Placental tissues from 15 patients with PE (≥ 32 weeks gestation) and 15 women with normal pregnancies (control group) were collected from the hospital. After delivery of the placenta, the tissue (0.5 × 0.5 × 0.5 cm) in the center of the placenta was quickly cut to avoid calcification points. Immediately after collection, the samples were snap‐frozen in liquid nitrogen and preserved at −80°C until analysis. The study protocol was approved by the Ethics Committees. Prior to sample collection, each patient provided written informed consent for participation in the study.

### 2.2 Cell Culture and Transfection

HTR‐8/SVneo (CL‐0765) was purchased from Pricella Biotechnology Co. Ltd. (Wuhan, China). The cells were cultured in RPMI‐1640 supplemented with 10% fetal bovine serum (FBS) and 1% penicillin–streptomycin at 37°C with 5% CO_2_. The cells were treated with a PI3K inhibitor (LY294002, 20 mM, Sigma, St. Louis, MO, USA) or activator (SC79) for 48 h. For cell transfection, shRNAs targeting the UBE3A gene (sh‐UBE3A‐1, sh‐UBE3A‐2, and sh‐UBE3A‐3) and the ITGB1 gene (sh‐ITGB1‐1, sh‐ITGB1‐2, and sh‐ITGB1‐3) were synthesized by GenePharma (Shanghai, China). Additionally, a negative control (sh‐NC) was synthesized. The UBE3A gene was synthesized and cloned into a pcDNA3.1 expression vector (GenePharma, Shanghai, China) to construct a UBE3A‐overexpressing vector (oe‐UBE3A). Following the manufacturer's protocol, transfection was carried out with Lipofectamine 2000 (Thermo Fisher Scientific, Waltham, MA, USA) for 48 h.

### 2.3 Quantitative Real‐Time PCR

Total RNA was extracted from tissues and cells with TRIzol reagent (Invitrogen, Carlsbad, CA, USA). cDNA synthesis was subsequently performed with the PrimeScript RT reagent Kit (Takara, Kyoto, Japan) to transcribe the RNAs. Real‐time PCR was conducted on the 7300 Real‐Time PCR system of Applied Biosystems using FastStart Universal SYBR Green Master Mix (Takara) for detection. The following primers were used: UBE3A F: 5′‐CTCGGGGTGACTACAGGAGA‐3′, R: 5′‐GGCAGAGGTGAAGCGTAAGT‐3′; ITGB1 F: 5′‐ATCCCAGAGGCTCCAAAGAT‐3′, R: 5′‐CCCCTGATCTTAATCGCAAA‐3′. The relative expression of mRNA was assessed using the 2^−∆∆Ct^ method, with GAPDH as an internal control.

### 2.4 Western Blotting

Cells were lysed with RIPA buffer (Beyotime, Shanghai, China). The protein concentrations were measured with a BCA kit (Beyotime). The proteins were transferred to PVDF membranes (Millipore, Bedford, MA, USA) after separation by 10% SDS‐PAGE, and then, the membranes were incubated with 5% skim milk for 1 h. The membranes were incubated with antibodies targeting the following proteins overnight at 4°C: UBE3A (ab272168, 1:1000, Abcam), ITGB1 (PA5‐78028, 1:1000, Invitrogen), PI3K (#3811, 1:1000, Cell Signaling Technology, CST, Danvers, MA, USA), phosphorylated PI3K (p‐PI3K, PA5‐104853, 1:1000, Invitrogen), AKT (#9272, 1:1000, CST), and phosphorylated AKT (p‐AKT, #9271, 1:1000, CST). The membranes were subsequently incubated with HRP‐conjugated secondary antibodies (#7074, 1:1000, CST) for 1 h. An ECL detection kit (Bio‐Rad, Hercules, CA, USA) was used to detect protein signals. The gray values of the bands were analyzed utilizing ImageJ (National Institutes of Health, NIH, Bethesda, MD, USA). GAPDH was used as a reference.

### 2.5 Immunohistochemistry (IHC)

To validate the expression of UBE3A and ITGB1 in the tissues of PE patients, the tissues were fixed in 10% formalin and sectioned into 4‐μm‐thick slices, which were then mounted on slides. Following xylene dewaxing, the sections were hydrated with various concentrations of ethanol (100%, 95%, 85%, and 75%) and subjected to antigen retrieval at 95°C. H_2_O_2_ was subsequently applied to inhibit endogenous peroxidase activity (0.3%, at room temperature), followed by incubation with an anti‐UBE3A primary antibody (ab290641, Abcam) and an anti‐ITGB1 primary antibody (PA5‐78028, Invitrogen) overnight at 4°C. The samples were further exposed to secondary antibodies for 30 min and then stained with diaminobenzidine (DAB) solution, followed by hematoxylin counterstaining, dehydration, and blocking with a gradient of ethanol concentrations (75%, 85%, 95%, and 100%). The resulting images were captured using a microscope (Olympus, Tokyo, Japan).

### 2.6 Cell Counting Kit‐8 (CCK‐8) Assay

A CCK‐8 kit (Beyotime) was used to assess cell viability. First, the cells were plated in 96‐well plates (1 × 10^5^ cells/mL) and cultured for 24 and 48 h. Subsequently, each well was treated with 10 μL of CCK‐8 solution and further incubated for 4 h. Thereafter, absorbance readings at 450 nm were measured with a microplate reader (Bio‐Rad).

### 2.7 Wound Healing Assay

Cells in each group in the logarithmic phase of growth were uniformly seeded into 6‐well plates. When the cells reached approximately 90% confluence, a uniform straight line was generated with the tip perpendicular to the culture plate. The cells were washed with phosphate‐buffered saline (PBS) three to five times and cultured for 24 h under the same conditions. Cell wounds were observed utilizing an inverted microscope (Olympus) and photographed. The distance of cell front movement and the width of the wounds were measured to analyze cell migration.

### 2.8 Transwell Assay

Cells (1 × 10^5^) in 200 μL of serum‐free culture medium were seeded in the upper chamber of a Transwell plate (8 μm; Millipore) that was precoated with Matrigel. Subsequently, 500 μL of DMEM supplemented with 10% serum was added to the bottom chamber. After a 24‐h incubation period, the cells in the chamber were fixed with 4% paraformaldehyde and stained with 0.1% crystal violet. The number of cells that invaded was determined by observing five random fields using a microscope (Olympus).

### 2.9 Flow Cytometry

Cells were collected 48 h after transfection. The cell concentration was adjusted to 5 × 10^6^ cells/mL. A total of 100 μL of the cell suspension was added to the bottom of the flow tube and incubated in the dark for 5 min with 10 μL of Annexin V‐FITC (Vazyme Biotech Co. Ltd., Nanjing, China). Next, 5 μL of propidium iodide (PI, Vazyme Biotech Co. Ltd.) solution and 400 μL of 1× binding buffer were added and mixed gently. We subsequently used a BD FACS flow cytometer (BD Biosciences, Franklin Lakes, NJ, USA) to assess apoptosis. The data were assessed utilizing FlowJo software (Tree Star Inc., San Carlos, CA, USA).

Cell cycle analysis was conducted following the guidelines provided by the manufacturer (Cell Cycle Analysis Kit, Kaiji, China). The cells were collected, washed with cold PBS, fixed with 70% ethanol at 4°C for 24 h, filtered through 300‐mesh nylon, and subsequently treated with 100 μL of RNase A in a 37°C water bath for 30 min. After incubation with PI in the dark for 30 min at 4°C, the cell cycle distribution was evaluated by a BD FACS flow cytometer (BD Biosciences). FlowJo software (Tree Star Inc.) was used for data analysis.

### 2.10 Coimmunoprecipitation (Co‐IP)

The cells were cultured in IP lysis buffer (Beyotime) for 30 min. Subsequently, the protein samples (500 μg) were exposed to 3 μg of an anti‐UBE3A antibody (ab272168, Abcam), an anti‐ITGB1 antibody (PA5‐78028, Invitrogen), or a control IgG (R5130, Sigma) and gently rotated at 4°C for 2 h. Following an overnight incubation at 4°C with 40 μL of protein‐A/G agarose beads (Roche, Basel, Switzerland), the beads were subjected to four wash cycles with IP lysis buffer. The samples were subsequently reconstituted in 40 μL of 2× SDS sample buffer and incubated at 95°C for 10 min. Immunoblotting was subsequently conducted to evaluate the samples.

### 2.11 Cycloheximide (CHX) Treatment

The cell culture medium was discarded and replaced with 1 mL of complete medium. A total of 500 μg of CHX (Sigma) was added to a 0.6 mL test tube. Next, 10 μL of DMSO was added to the tube and mixed (the final concentration of CHX was 50 mg/mL). It is better to use freshly dissolved CHX to ensure maximum activity. One microliter of CHX was slowly added to 1 mL of cell culture medium. The plate was gently shaken so that it was evenly mixed. Similarly, 1 μL of DMSO was added to the corresponding control wells. Then, the cells were cultured for different times; the ITGB1 protein half‐life was analyzed.

### 2.12 Detection of ITGB1 Ubiquitination Levels

Cells were transfected with MYC‐ITGB1 for 24 h. Next, the transfected cells were pretreated with 30 μM MG132 (Sigma) for 6 h, lysed, and incubated with an anti‐MYC antibody (PA5‐85185, Invitrogen) conjugated to agarose beads (Roche) overnight at 4°C. After washing, the beads were boiled with SDS loading buffer. The protein complexes that had been subjected to immunoprecipitation were subsequently examined by Western blotting analysis with an antiubiquitin antibody (13–1600, Invitrogen).

### 2.13 Statistical Analysis

The data were analyzed with GraphPad Prism 8.0 (GraphPad Prism; La Jolla, CA, USA) and are presented as the means ± standard deviations (SDs). Statistical differences between two groups were determined with Student's *t* test. Data among multiple groups were compared with one‐way ANOVA. *p* < 0.05 was considered to indicate a statistically significant difference.

## 3 Results

### 3.1 Detection of UBE3A and ITGB1 Expression in Placental Tissues of PE Patients

**FIGURE 1:**
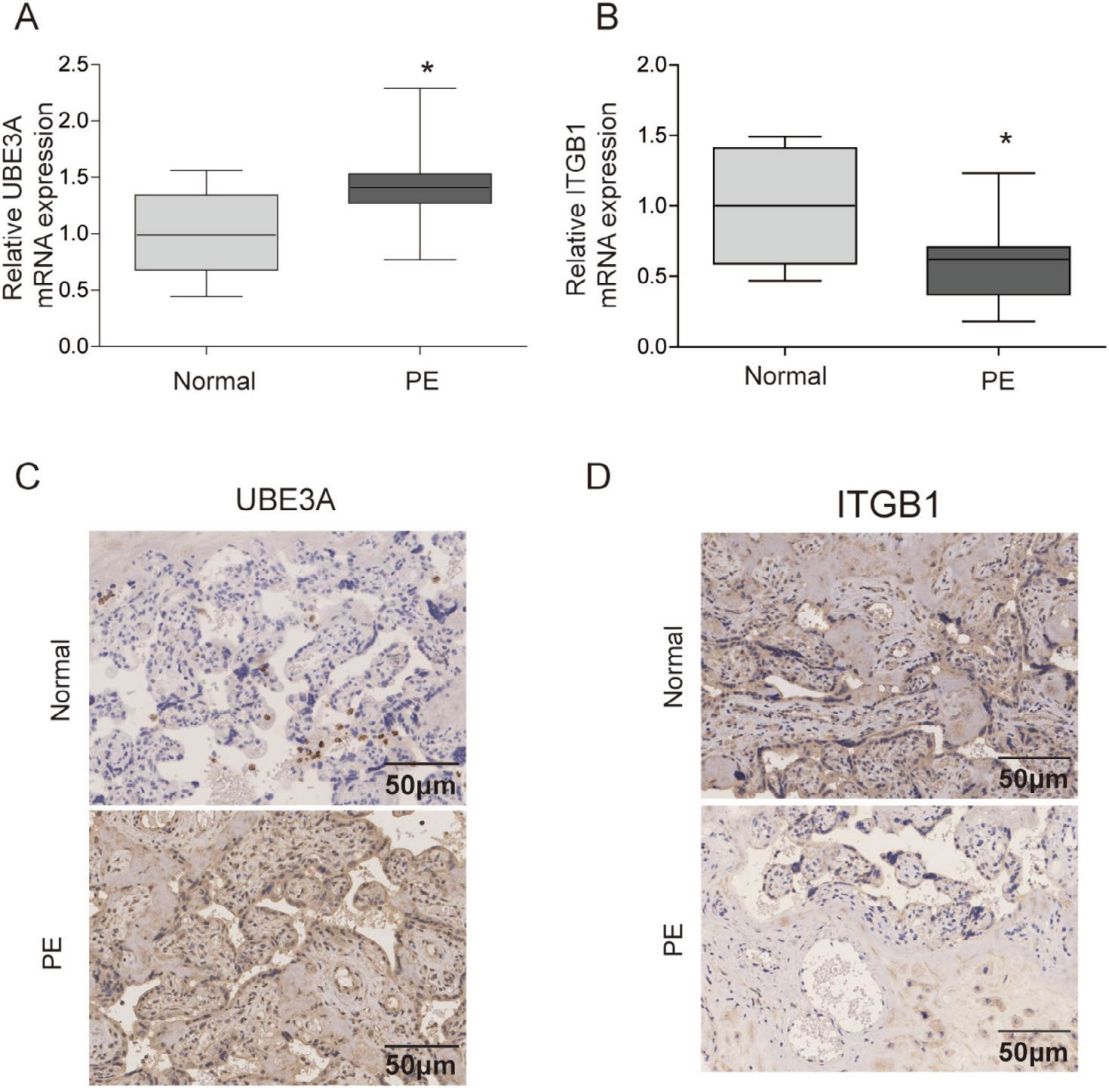
Detection of UBE3A and ITGB1 expression in the placental tissues of PE patients. Placental tissues of PE patients were extracted for analysis. (A) UBE3A mRNA expression was measured by RT‐qPCR. (B) The ITGB1 mRNA level was measured by RT‐qPCR. (C) The protein expression of UBE3A was evaluated by IHC. (D) The protein expression of ITGB1 was assessed with IHC. The values are expressed as the mean ± SD of three separate determinations. *p < 0.05.

First, we measured the mRNA levels of UBE3A and ITGB1 in the placental tissues of PE patients. Compared with that in normal tissues, the mRNA expression of UBE3A was upregulated, and the mRNA expression of ITGB1 was downregulated in PE patients (Figure 1A,B). Furthermore, IHC staining revealed that the UBE3A protein level was increased in PE patients, whereas the ITGB1 level was decreased (Figure 1C,D).

### 3.2 UBE3A Knockdown Promoted HTR‐8/SVneo Cell Migration and Invasion and Inhibited Apoptosis

**FIGURE 2:**
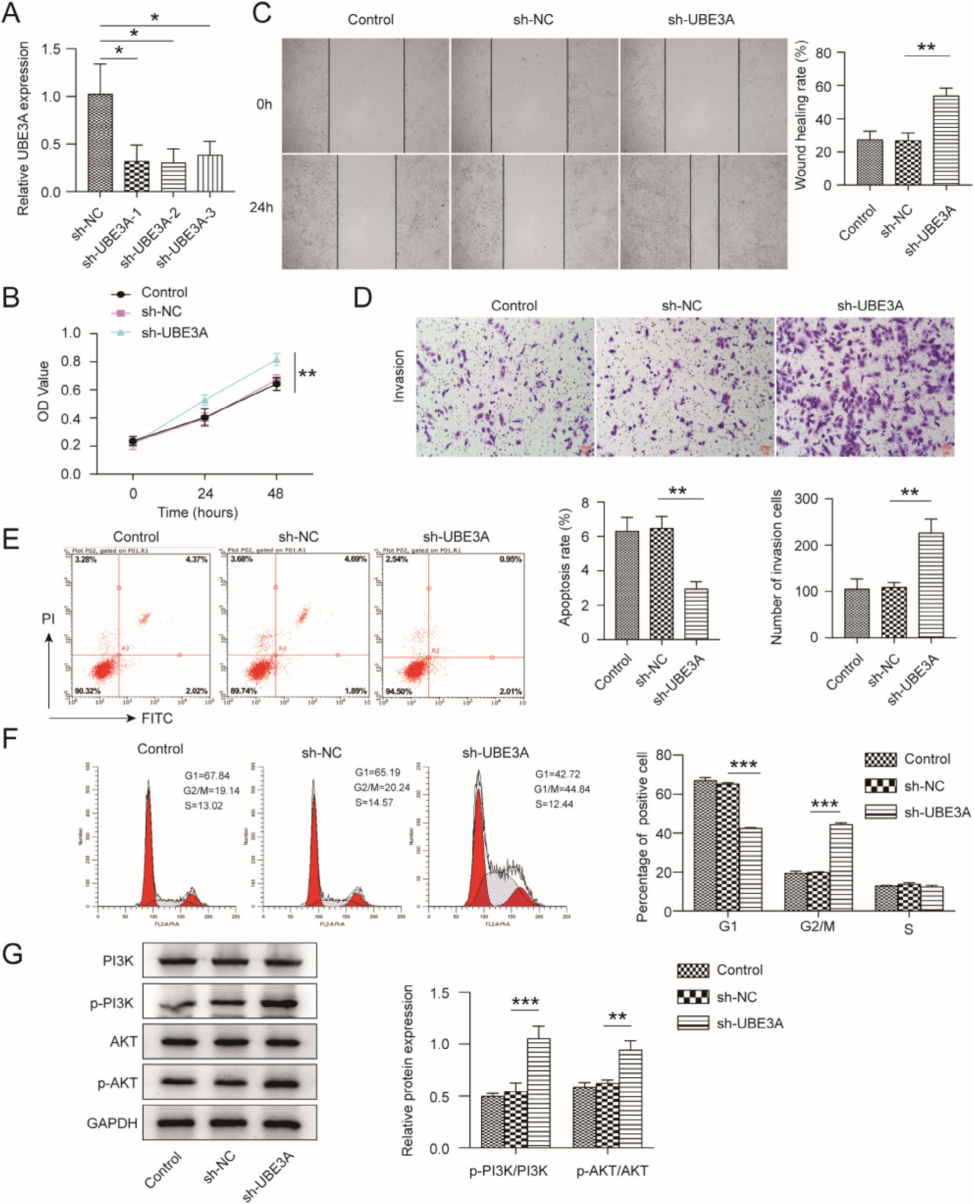
UBE3A knockdown promoted HTR‐8/SVneo cell migration and invasion and inhibited apoptosis. HTR‐8/SVneo cells were transfected with sh‐NC or sh‐UBESA. (A) RT‐qPCR was used to measure the knockdown efficiency of UBE3A. (B) The effect of UBE3A knockdown on cell viability was analyzed with a CCK‐8 assay. (C and D) The effects of UBE3A knockdown on cell migration and invasion were assessed with wound healing and Transwell assays. (E and F) The effects of UBE3A depletion on apoptosis and cell cycle progression were analyzed with flow cytometry. (G) The effects of UBE3A silencing on PI3K/AKT signaling were determined by Western blotting. The values are expressed as the mean ± SD of three separate determinations. *p < 0.05, **p < 0.01, and ***p < 0.001.

HTR‐8/SVneo cells can be utilized to study trophoblast and placental biology [15]. To study the effect of UBE3A knockdown on the biochemical function of HTR‐8/SVneo cells, we designed three shRNAs to knock down UBE3A and transfected them into HTR‐8/SVneo cells. The expression of UBE3A was significantly reduced by sh‐UBE3A transfection (Figure 2A). Subsequently, the results of the CCK‐8 analysis indicated that cell viability was increased in the sh‐UBE3A group (Figure 2B). In addition, wound healing and Transwell assays demonstrated that the migration and invasion of cells were increased in the sh‐UBE3A group (Figure 2C,D). The flow cytometry results revealed that the level of apoptosis in the sh‐UBE3A group was decreased, and more cells remained in the G2 phase, suggesting that UBE3A knockdown inhibited apoptosis (Figure 2E,F). Increasing evidence has revealed the relationship between PE and the PI3K/AKT signaling pathway [14]. We further verified the effect of UBE3A knockdown on the PI3K/AKT signaling pathway by Western blotting. The levels of phosphorylated PI3K and AKT were increased after UBE3A was silenced (Figure 2G). HTR‐8/SVneo cells were subsequently transfected with oe‐NC or oe‐UBE3A. UBE3A overexpression inhibited HTR‐8/SVneo cell migration and invasion, promoted apoptosis, and decreased the levels of phosphorylated PI3K and AKT (Figure S1). These data suggest that UBE3A knockdown promotes HTR‐8/SVneo cell migration and invasion, inhibits apoptosis, and activates the PI3K/AKT signaling pathway.

### 3.3 UBE3A Promoted the Ubiquitination of ITGB1

**FIGURE 3:**
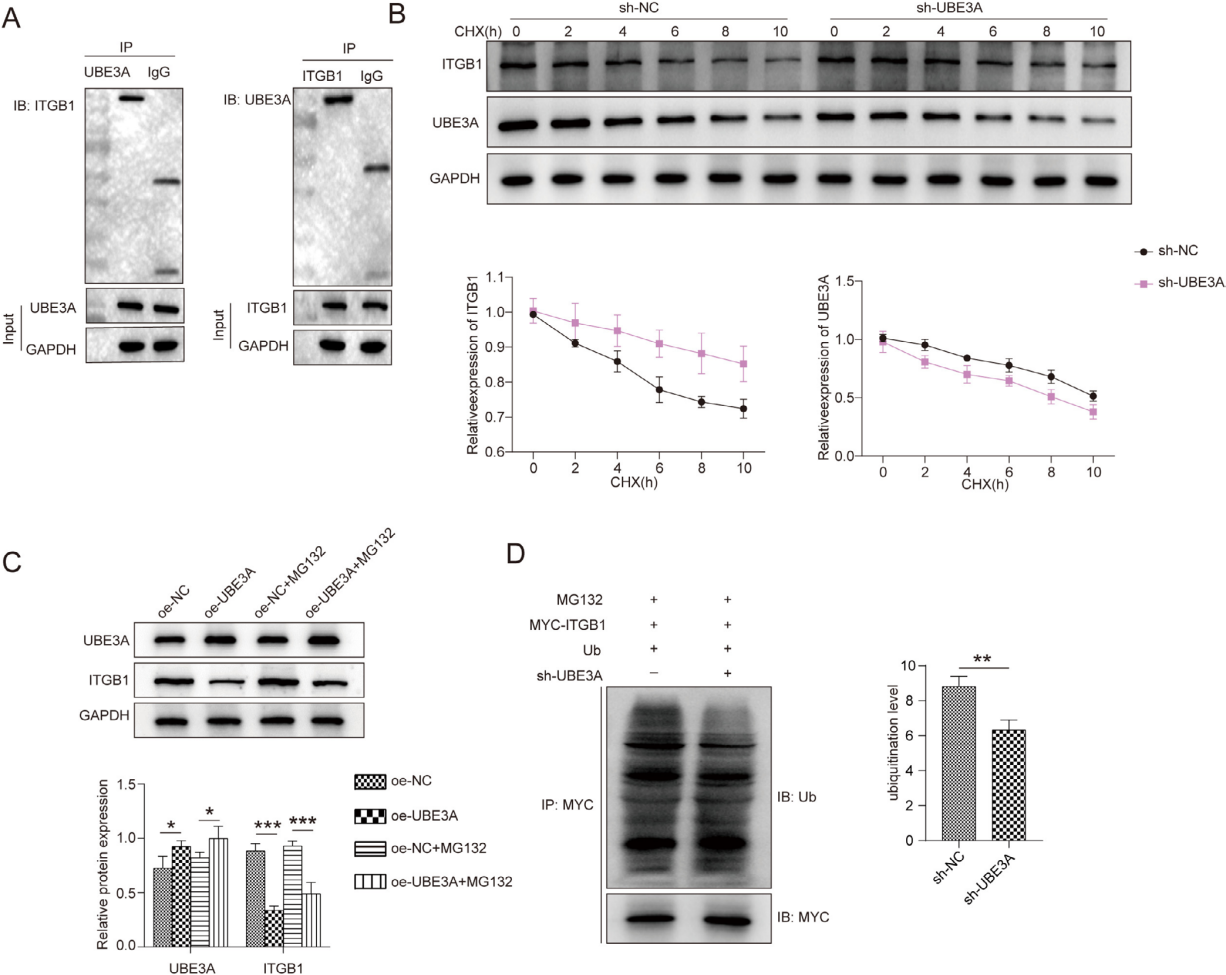
UBE3A promoted the ubiquitination of ITGB1. (A) Co‐IP confirmed the binding of UBE3A to the ITGB1 protein in HTR‐8/SVneo cells. (B) HTR‐8/SVneo cells transfected with sh‐NC or sh‐UBE3A were incubated with 100 μg/mL CHX for 0, 2, 4, 6, 8, or 10 h. Western blotting was used to measure the stability of the ITGB1 protein. (C) HTR‐8/SVneo cells were divided into different groups: oe‐NC, oe‐UBE3A, oe‐NC + MG132 (proteasome inhibitor), and oe‐UBE3A + MG132. The expression of UBE3A and ITGB1 was evaluated by Western blotting. (D) The level of ubiquitinated ITGB1 was evaluated using Co‐IP. All the experiments were repeated three times, and the error bars indicate the mean ± SD. *p < 0.05, **p < 0.01, and ***p < 0.001.

According to bioinformatics predictions, the E3 ubiquitin ligase UBE3A potentially plays a crucial role in trophoblast cell migration and invasion by mediating ITGB1 ubiquitination. Therefore, we further studied the relationship between UBE3A and ITGB1. Co‐IP confirmed that UBE3A binds to the ITGB1 protein in HTR‐8/SVneo cells (Figure 3A). After treatment with the protein synthesis inhibitor CHX, UBE3A downregulation reduced ITGB1 protein degradation (Figure 3B). Furthermore, the Western blot results revealed that UBE3A overexpression increased UBE3A protein levels and decreased ITGB1 protein levels, whereas the addition of MG132 (a proteasome inhibitor) increased ITGB1 protein expression in cells that were transfected with oe‐UBE3A; these results suggested that the degradation of ITGB1 by UBE3A is mediated by the proteasome pathway (Figure 3C). Next, we found that the knockdown of UBE3A reduced the level of ubiquitinated ITGB1 (Figure 3D). Thus, these results indicate that UBE3A leads to ITGB1 degradation by promoting ITGB1 ubiquitination.

### 3.4 UBE3A knockdown Promoted HTR‐8/SVneo Cell Migration and Invasion and Inhibited Apoptosis by Influencing the ITGB1/PI3K/AKT Axis

**FIGURE 4:**
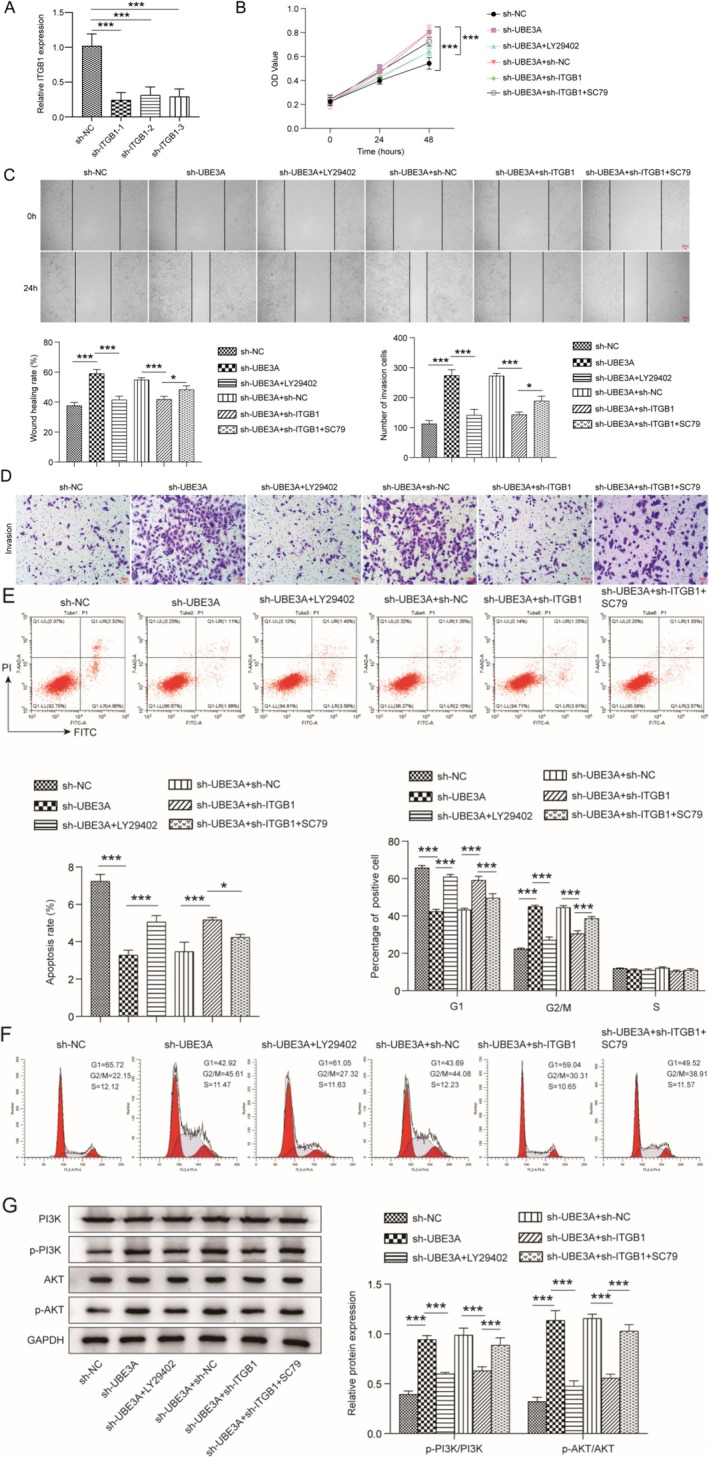
UBE3A inhibited HTR‐8/SVneo cell migration and invasion and promoted apoptosis by influencing the ITGB1/PI3K/AKT axis. (A) Verification of the knockdown efficiency of ITGB1. Next, HTR‐8/SVneo cells were transfected with sh‐UBEA and sh‐ITGB1 and then treated with LY294002 (PI3K inhibitor) or SC79 (PI3K/AKT activator). (B) A CCK‐8 assay was used to analyze the effect of UBE3A on cell viability by regulating the ITGB1‐mediated PI3K/AKT signaling pathway. (C and D) Wound healing and Transwell assays were used to analyze the effects of UBE3A on cell migration and invasion through the ITGB1‐mediated PI3K/AKT signaling pathway. (E and F) Flow cytometry was used to analyze the effects of UBE3A on apoptosis and cell cycle progression by regulating the ITGB1‐mediated PI3K/AKT signaling pathway. (G) The effects of UBE3A knockdown and ITGB1 knockdown on the PI3K/AKT signaling pathway were assessed by Western blotting. The values are expressed as the mean ± SD of three separate determinations. *p < 0.05 and ***p < 0.001.

A recent study revealed that ITGB1 overexpression in HTR‐8/SVneo cells can increase cell proliferation, migration, and invasion, decrease cell apoptosis, and increase the levels of phosphorylated PI3K and AKT [14]. To further explore the effects of UBE3A on ITGB1 and downstream signaling pathways, we performed experiments with sh‐UBE3A and sh‐ITGB1. RT‐qPCR analysis revealed that sh‐ITGB1 could reduce the mRNA expression of ITGB1 (Figure 4A). Compared with that of the sh‐NC group, cell viability was increased by sh‐UBE3A. Although ITGB1 downregulation and LY294002 (a PI3K inhibitor) treatment suppressed cell viability in sh‐UBE3A‐transfected cells, the cell viability was restored by the PI3K/AKT activator SC79 (Figure 4B). Additionally, cell migration and invasion were promoted by sh‐UBE3A, and the introduction of sh‐ITGB1 or LY294002 reduced migration and invasion; however, this effect was reversed by SC79 in cells that had been co‐transfected with sh‐UBE3A and sh‐ITGB1 (Figure 4C,D). Then, we performed flow cytometry analysis. We found that UBE3A silencing significantly inhibited apoptosis and resulted in more cells remaining in the G2 phase, and these effects were reversed by ITGB1 depletion or LY294002 treatment; however, the effect of sh‐ITGB1 on sh‐UBE3A‐treated cells was reversed by SC79 treatment (Figure 4E,F). Finally, the levels of p‐PI3K and p‐AKT were increased by sh‐UBE3A, and this effect was reversed by sh‐ITGB1 or LY294002; however, the introduction of SC79 increased the levels of p‐PI3K and p‐AKT in cells that were co‐transfected with sh‐UBE3A and sh‐ITGB1 (Figure 4G). In conclusion, UBE3A inhibits cell migration and invasion and promotes apoptosis by modulating ITGB1 and mediating the PI3K/AKT signaling pathway.

## 4 Discussion

PE is an idiopathic transient disease that occurs during pregnancy. PE involves multiple organ and system injuries, which can seriously threaten the safety of mothers and infants throughout the perinatal period [16]. Because the pathogenesis of PE is not clear, specific treatments are lacking. Researchers have found that the abnormal migration and invasion of human trophoblast cells are crucial in the development of PE [17]. Therefore, it is very important to study the mechanism underlying trophoblast cell migration and invasion, with the aim of identifying novel molecular targets for PE treatment. Our results confirmed, for the first time, that UBE3A inhibited the activation of PI3K/AKT signaling by promoting ITGB1 ubiquitin‐proteasome degradation, thereby inhibiting the migration and invasion of trophoblastic cells. This suggests that UBE3A overexpression may be related to the progression of PE.

Ubiquitination plays multiple roles in trophoblastic cell dysfunction and PE. For example, DOCK1 is implicated in the regulation of DUSP4 ubiquitin levels, which may further regulate the function of extravillous trophoblasts (EVTs) through the regulation of E3 ligase HUWE1 [18]. During PE, Thrombospondin‐1 regulates trophoblast necroptosis through NEDD4‐mediated TAK1 ubiquitination [9]. Abnormal expression of E6AP (encoded by UBE3A) contributes to the development of two neurodevelopmental conditions: Angelman syndrome and autism [19]. Increased expression of UBE3A has previously been reported in the placental tissue of PE patients. In addition, miR‐218‐5p targets UBE3A expression to block ubiquitin‐mediated SATB1 degradation, promote trophoblast infiltration, and alleviate endoplasmic reticulum/oxidative stress [11]. When UBE3A was deleted, cell cultures of MEFs exhibited enhanced proliferation together with reduced apoptosis [20]. G1/G2 phase arrest is a “buffer period” for cells to gain time for repair, and its failure triggers apoptosis [20, 21]. In addition, G2 phase arrest plays a protective role in cell injury [22]. Our results confirmed that UBE3A was upregulated in the placentas of PE patients. We also proved that downregulation of UBE3A promoted the migration and invasion of HTR‐8/SVneo cells. The level of apoptosis in the UBE3A knockdown group was decreased, and more cells remained in the G2 phase, suggesting that UBE3A depletion suppressed apoptosis. These results indicated a potentially significant role for UBE3A in the progression of PE.

ITGB1 plays an important role in HTR‐8/SVneo cells and PE. Through ALK4‐mediated SMAD2/3‐SMAD4 pathway activation, ITGB1 facilitates trophoblast invasion enhanced by activin A [23]. MiR‐134 represses trophoblast invasion in PE by downregulating ITGB1 expression [13]. ADAM12S promotes trophoblast migration by inducing ITGB1‐mediated cellular spreading [24]. These studies suggest that the regulation of ITGB1 may affect the development of PE. UBE3A is an E3 ligase that is involved in substrate recognition and ubiquitination. Moreover, UBE3A controls the ubiquitination and breakdown of PTPA to govern the function of PP2A and the shape of dendritic spines [25]. UBE3A regulates the function and synaptic plasticity of mTORC1 by regulating the ubiquitination and degradation of p18/LAMTOR1 [26]. However, researchers have not reported a relationship between UBE3A and ITGB1. Here, our findings revealed that ITGB1 expression was downregulated in the placental tissues of PE patients. Importantly, we demonstrated for the first time that UBE3A interacted with ITGB1 in HTR‐8/SVneo cells and that UBE3A could induce ITGB1 degradation to promote PE progression.

The PI3K/AKT signaling pathway plays a role in regulating the proliferation, migration, and invasion of trophoblast cells. For example, hsa_circ_0088196 suppresses the migration and invasion of trophoblast cells through the PI3K/AKT pathway [27]. STX2 promotes the growth, migration, and invasion of trophoblast cells by activating the PI3K/AKT pathway in PE [28]. CircLRRK1 inhibits the proliferation, migration, and invasion of trophoblast cells by targeting miR‐223‐3p via the PI3K/AKT signaling pathway [29]. MiR‐187 controls the proliferation, migration, and invasion of trophoblast cells by suppressing BCL6‐induced PI3K/AKT activation [30]. Notably, integrin β1 controls the proliferation, apoptosis, and migration of trophoblast cells by activating PI3K/AKT [14]. Here, our results confirmed, for the first time, that UBE3A inhibited the migration and invasion of trophoblast cells and promoted apoptosis by modulating ITGB1‐mediated PI3K/AKT signaling, suggesting that UBE3A might promote the development of PE by inhibiting the PI3K/AKT signaling pathway.

In general, we demonstrated that UBE3A was upregulated in the placental tissues of PE patients, whereas ITGB1 was downregulated. By regulating the ITGB1‐mediated PI3K/AKT signaling pathway, UBE3A inhibited the migration and invasion of trophoblast cells, promoted apoptosis, and accelerated the development of PE. Our study links UBE3A and ITGB1 for the first time, providing a novel target for the clinical treatment of PE. In the future, the molecular mechanisms related to the UBE3A/ITGB1/PI3K/AKT axis need to be further verified in animal models.

## Conflicts of Interest

The authors declare no conflicts of interest.

## Supporting information


**Figure S1:** UBE3A overexpression inhibited HTR‐8/SVneo cell migration and invasion and promoted apoptosis. HTR‐8/SVneo cells were transfected with oe‐NC or oe‐UBE3A. (A) RT‐qPCR was used to determine the transfection efficiency of oe‐UBE3A. (B) The effect of UBE3A overexpression on cell viability was analyzed with a CCK‐8 assay. (C and D) The effects of UBE3A overexpression on cell migration and invasion were assessed with wound healing and Transwell assays. (E and F) The effects of UBE3A overexpression on apoptosis and cell cycle progression were analyzed utilizing flow cytometry. (G) The effects of UBE3A overexpression on PI3K/AKT signaling were determined by Western blotting. The values are expressed as the mean ± SD of three separate determinations. *p < 0.05, **p < 0.01, and ***p < 0.001.

## Data Availability

All data generated or analysed during this study are included in this article. The datasets used and/or analysed during the current study are available from the corresponding author on reasonable request.
