# Supplementary figures and images for: UBE3A Inhibits Trophoblast Cell Migration and Invasion by Promoting ITGB1 Degradation and Affecting PI3K/AKT Signaling
Source: Kaohsiung J Med Sci. 2025 Oct 15;42(4):e70122. doi: 10.1002/kjm2.70122 (PMC13147960; doi:10.1002/kjm2.70122)

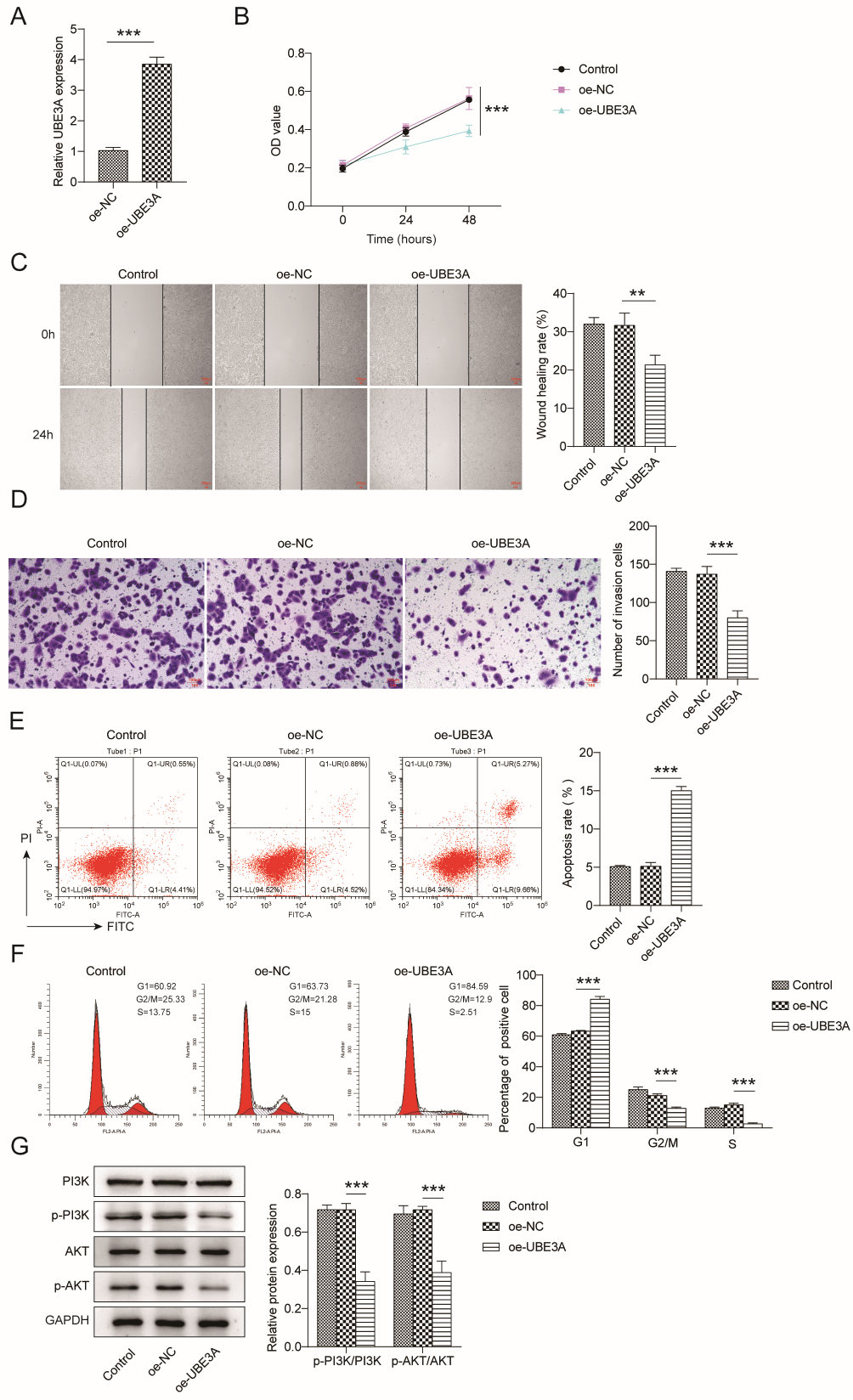

Supplement: Supplementary file 1 — Figure S1: UBE3A overexpression inhibited HTR‐8/SVneo cell migration and invasion and promoted apoptosis. HTR‐8/SVneo cells were transfected with oe‐NC or oe‐UBE3A. (A) RT‐qPCR was used to determine the transfection efficiency of oe‐UBE3A. (B) The effect of UBE3A overexpression on cell viability was analyzed with a CCK‐8 assay. (C and D) The effects of UBE3A overexpression on cell migration and invasion were assessed with wound healing and Transwell assays. (E and F) The effects of UBE3A overexpression on apoptosis and cell cycle progression were analyzed utilizing flow cytometry. (G) The effects of UBE3A overexpression on PI3K/AKT signaling were determined by Western blotting. The values are expressed as the mean ± SD of three separate determinations. *p < 0.05, **p < 0.01, and ***p < 0.001. [file KJM2-42-e70122-s001.tiff]
